# Prickle isoforms control the direction of tissue polarity by microtubule independent and dependent mechanisms

**DOI:** 10.1242/bio.016162

**Published:** 2016-02-10

**Authors:** Katherine A. Sharp, Jeffrey D. Axelrod

**Affiliations:** 1 Department of Pathology, Stanford University School of Medicine, 300 Pasteur Drive, L235, Stanford, CA 94305, USA; 2 Department of Genetics, Stanford University School of Medicine, 300 Pasteur Drive, L235, Stanford, CA 94305, USA

**Keywords:** Planar cell polarity, Prickle, Spiny-legs, *Drosophila*, Fat

## Abstract

Planar cell polarity signaling directs the polarization of cells within the plane of many epithelia. While these tissues exhibit asymmetric localization of a set of core module proteins, in *Drosophila*, more than one mechanism links the direction of core module polarization to the tissue axes. One signaling system establishes a polarity bias in the parallel, apical microtubules upon which vesicles containing core proteins traffic. Swapping expression of the differentially expressed Prickle isoforms, Prickle and Spiny-legs, reverses the direction of core module polarization. Studies in the proximal wing and the anterior abdomen indicated that this results from their differential control of microtubule polarity. Prickle and Spiny-legs also control the direction of polarization in the distal wing (D-wing) and the posterior abdomen (P-abd). We report here that this occurs without affecting microtubule polarity in these tissues. The direction of polarity in the D-wing is therefore likely determined by a novel mechanism independent of microtubule polarity. In the P-abd, Prickle and Spiny-legs interpret at least two directional cues through a microtubule-polarity-independent mechanism.

## Abstract (precis)

**Summary:** Learning how Pk and Sple differentially interpret directional cues allows a broader understanding of the varied signals and mechanisms linking the core module to tissue axes in planar cell polarity.

## INTRODUCTION

Planar cell polarity (PCP) is the alignment of cells within the plane of an epithelium orthogonal to the apicobasal axis. PCP signaling provides cells with spatial information that they use to determine the direction in which polarized structures grow or to direct cell migration during tissue remodeling (Devenport, 2014; Zallen, 2007). PCP has been most extensively studied in the fruit fly *Drosophila melanogaster* where it is required for the proper orientation of morphological features such as the small trichomes (hairs) that emerge from cells of the wings and body of the adult fly (Devenport, 2014; Zallen, 2007). Many genes that govern PCP in *Drosophila* are known, and while these genes are largely conserved in vertebrates where PCP is increasingly being studied (Carroll and Yu, 2012; Ezan and Montcouquiol, 2013; Sokol, 2015; Vladar et al., 2009), *Drosophila* continues to be a powerful system in which to study the cell biological mechanisms of PCP signaling.

A question central to PCP signaling is how the direction in which cells polarize with respect to the tissue axes is determined. PCP is governed by molecular signaling modules with specific functions (Axelrod, 2009; Tree et al., 2002b). The core module polarizes individual cells by establishing molecular asymmetry. A feature of the core module mechanism is the communication of polarity between neighboring cells to create areas of local alignment (Maung and Jenny, 2011; Vladar et al., 2009). However, the core module has no intrinsic mechanism to orient its action to the tissue axes and therefore additional directional input from tissue-wide or global signals is required. Models have been proposed in which global signaling modules act directly on the core module (Ambegaonkar and Irvine, 2015; Ayukawa et al., 2014; Harumoto et al., 2010; Ma et al., 2003; Matis et al., 2014; Olofsson et al., 2014), whereas other models suggest that global signals may provide directional information to cells independent of the core module (Casal et al., 2006; Donoughe and DiNardo, 2011). Studies probing the mechanism of core module function provided clues that led to the description of a mechanistic link between tissue-wide signals and core PCP proteins.

The core module in flies consists of two protein complexes which localize to opposite sides of the apical cortex of each cell: Frizzled (Fz), Dishevelled (Dsh) and Diego (Dgo) on one side and Van Gogh (Vang, aka Stbm) and Prickle (Pk; aka Prickle-Spiny-legs) on the other side (Devenport, 2014; Maung and Jenny, 2011; Vladar et al., 2009; Zallen, 2007). Flamingo (Fmi, aka Stan, an atypical cadherin) is found in both complexes (Devenport, 2014; Goodrich and Strutt, 2011). Fmi complexed with Fz homodimerizes selectively with Fmi complexed with Vang, thereby forming stable intercellular complexes that communicate their asymmetric accumulation between neighboring cells (Chen et al., 2008; Strutt and Strutt, 2007, 2008, 2009). At the cell cortex, intra- and inter-cellular interactions between core complex proteins produce bistability, amplifying small asymmetries to achieve robust locally aligned polarity (Ayukawa et al., 2014; Bastock et al., 2003; Cho et al., 2015; Feiguin et al., 2001; Jenny et al., 2003, 2005; Strutt et al., 2011; Tree et al., 2002a). In the fly wing and abdomen, the Fz complex accumulates to high levels distally (wing) or posteriorly (abdomen), while the Vang complex accumulates proximally (wing) or anteriorly (abdomen). This asymmetric localization of the core module proteins is required to restrict hair growth to the distal or posterior sides of wing or abdominal cells, respectively (reviewed in Carvajal-Gonzalez and Mlodzik, 2014; Devenport, 2014). While the core module allows neighboring cells to create local areas of alignment, alone it is lacking a connection to the tissue axis. A parallel network of non-centrosomal, apical microtubules has been observed to support directional vesicular trafficking of core complex components Fz and Dsh from one side of the cell to the other (Harumoto et al., 2010; Matis et al., 2014; Olofsson et al., 2014; Shimada et al., 2006), suggesting the possibility that this directional trafficking might provide a source of directional input bias.

In multiple tissues, one source of tissue-wide signaling is proposed to come from a global module consisting of Fat (Ft), Dachsous (Ds), and Four-jointed (Fj). Ds and Ft are atypical cadherins that form heterodimers across intercellular junctions (Ambegaonkar et al., 2012; Brittle et al., 2010, 2012; Hale et al., 2015; Matakatsu and Blair, 2004; Matis and Axelrod, 2013; Sharma and McNeill, 2013). Both Ft and Ds are phosphorylated by Fj, a Golgi associated ectokinase (Brittle et al., 2010; Hale et al., 2015; Ishikawa et al., 2008). Fj is expressed in a gradient along the proximal-distal axis, with high distal and low proximal expression (Matakatsu and Blair, 2004; Rogulja et al., 2008; Zeidler et al., 2000). Because phosphorylation by Fj makes Ds a worse ligand for Ft but Ft a better ligand for Ds (Brittle et al., 2010; Hale et al., 2015), the kinase activity of Fj helps to translate the Fj expression gradient into subcellular asymmetry of Ds-Ft heterodimers, with Ds accumulating on one side and Ft on the opposite side of each cell (Ambegaonkar et al., 2012; Brittle et al., 2010; Hale et al., 2015). Furthermore, while Ft is expressed uniformly, Ds is expressed in gradients opposite to those of Fj (Casal et al., 2002; Hogan et al., 2011; Ma et al., 2003; Matakatsu and Blair, 2004; Rogulja et al., 2008), with the imbalance of Ds expression also favoring the same orientation of Ft-Ds homodimers.

A potentially confounding feature of this model is that, in different tissues, the relationship between the direction of Ds and Fj gradients and the direction of core module polarization is inconsistent. In wing and posterior abdomen (P-abd), Fz accumulates on (and, hence, hairs grow towards) the side of cells toward the low end of the Ds gradient, while in eye and anterior abdomen (A-abd), Fz accumulates toward the high end of the Ds gradient (Casal et al., 2002, 2006; Olofsson et al., 2014). Thus, if both systems are providing directional information, a mechanism for reconciling these apparently opposite signals must exist. This inconsistency was reconciled by observations regarding the tissue specific activities of two protein isoforms of the *prickle* locus (Gubb et al., 1999), Prickle (Pk) and Spiny-legs (Sple) (Fig. S1A). Isoform specific mutations of Pk and Sple had shown that they are required in mutually exclusive tissues (Gubb et al., 1999; Lin and Gubb, 2009) where either one can fulfill the core module function. However, changing which isoform is expressed in a given tissue can reverse the polarity of that tissue (Ambegaonkar and Irvine, 2015; Ayukawa et al., 2014; Doyle et al., 2008; Lawrence et al., 2004; Lee and Adler, 2002; Lin and Gubb, 2009; Olofsson et al., 2014; Strutt et al., 2013). For example, Pk is normally expressed in the wing, but if Sple is expressed in its place the hairs in the wing will grow proximally instead of distally (Ayukawa et al., 2014; Olofsson et al., 2014; Strutt et al., 2013). Conversely, if Pk expression is substituted for the normally predominant Sple expression in the anterior compartment of the abdomen (A-abd), hairs in the A-abd will grow anteriorly instead of posteriorly (Olofsson et al., 2014).

This tissue polarity reversal is associated with the ability of Pk and Sple to control the polarity bias of the parallel, apical microtubules and therefore with the movement of Fz and Dsh vesicles, which traffic toward the microtubule plus-ends (Harumoto et al., 2010; Olofsson et al., 2014; Shimada et al., 2006). In both the wing and the A-abd, Pk biases microtubule plus-ends towards the side of the cell near the low-end of the Ds gradient, whereas Sple biases microtubule plus-ends towards the side of the cell near the high-end (Olofsson et al., 2014). Subtly increasing the amount of Dsh and Fz on one side of the cell (Harumoto et al., 2010; Matis et al., 2014; Olofsson et al., 2014; Shimada et al., 2006) is proposed to direct core complex polarization in a defined direction. Expression of Ft and Ds is required for the apical microtubules to form a parallel network: when *ft* or *ds* are mutated, the microtubules within cells become misaligned (Harumoto et al., 2010; Matis et al., 2014; Olofsson et al., 2014). Therefore, selective control of microtubule polarity by Pk or Sple provides a mechanism through which oppositely oriented Ds-Fj gradients could be interpreted in the same way by the core module in the wing and A-abd.

Another mechanism linking Ft-Ds organization to the direction of core PCP polarization was suggested by the observation that Ds, and its interacting partner Dachs (D), physically interact with the Sple isoform. It is hypothesized that Sple binding to Ds or D may anchor Sple to the Ds- and D-rich side of cells (Ambegaonkar and Irvine, 2015; Ayukawa et al., 2014). Anchoring Sple to one side could tether the Vang-Sple complex to that side, and might be a way of determining the direction of core module polarization. This mechanism would only explain polarization in Sple-dependent tissues, since Pk does not bind to Ds or D. Whether Pk-dependent tissues might use a corresponding mechanism such as Pk binding and anchoring to another protein is not known. Anchoring Sple to one side of cells via Ds and D binding could provide a directional cue to core module polarization either in addition to or independent of microtubule polarity. Indeed, anchoring Sple might be the mechanism by which Sple biases microtubule polarity in the opposite direction as Pk.

In addition to the Ft-Ds-Fj module, there are evidently other tissue level signals that help to orient cells with respect to the tissue axes in the wing. This is apparent when the Ft-Ds-Fj system is disabled either in clones or in the entire tissue. Whereas global polarity is lost in the proximal, central region of the wing (P-wing), resulting in swirling polarity patterns, in the distal, peripheral regions of the wing (D-wing) polarity remains largely unaffected, suggesting additional cues are derived from the wing periphery (Matis et al., 2014). Notably, the proximal/central, Ft-Ds-Fj dependent region corresponds to the region of the wing in which microtubule plus-ends are biased distally. In contrast, microtubules do not display a proximal-distal plus-end bias in the D-wing (Harumoto et al., 2010). Thus, some other signal is likely providing directional information to cells in the D-wing.

Wnt ligands are a proposed source for this signal. It has been observed that overexpressing Wnt4 in a clonal patch of cells will cause surrounding cells to reorient and grow hairs pointing towards the clone (Lim et al., 2005; Wu et al., 2013). Additionally, Wnt4 is expressed at the wing margin (Lim et al., 2005; Wu et al., 2013) and thus could be a signal that orients cells towards the margin and distal end of the wing. *wnt4* mutant wings do not show a PCP phenotype; however, a PCP phenotype was observed in wings mutant for both *wnt4* and *wg*. This phenotype was proposed to be mediated through effects on Fz and Vang, though the mechanism for the action of Wnt4 and Wg in PCP remains elusive (Wu et al., 2013). In the dorsal abdomen, making clones of cells simultaneously mutant for four of the seven *Drosophila wnt* genes (*wg*, and *wnt4*, *6*, and *10*) does not disrupt PCP (Lawrence et al., 2002), and while a long range signal in addition to Ft-Ds-Fj has been hypothesized in this tissue, a candidate for this signal has not been identified (Lawrence et al., 2002).

It is interesting to observe that while the P-wing is sensitive to Ft and Ds and the D-wing is sensitive to Wnt4 and Wg, nearly the entire wing responds to isoform swapping from the *prickle* locus to orient hair polarity. Loss of *sple* expression does not perturb polarity, yet when Sple is overexpressed, hairs in all but a few cells at the wing margin are reversed and grow proximally (Doyle et al., 2008; Olofsson et al., 2014; Strutt et al., 2013).

Thus, while a coherent set of rules can explain the relationship between Ft-Ds-Fj, Pk/Sple, microtubule polarity and the activity of the core module in the P-wing and A-abd, it appears that a different regime may operate in the D-wing. Furthermore, while Pk and Sple expression has been shown to control the direction of hair growth in the P-abd (Lawrence et al., 2004; Olofsson et al., 2014), the mechanisms at work in this region have not been explored in detail.

Here, we examine the signals that Pk and Sple respond to and the mechanisms they use to control the direction of tissue polarity in the D-wing and P-abd. We show that the direction of polarity in the D-wing and P-abd is determined by Pk and Sple without affecting microtubule polarity. Further, in the P-abd, we show that control of polarity by Pk and Sple requires Ft-Ds-Fj and another, cryptic signal. We put these tissues forward as tools for the discovery of additional mechanisms of tissue-wide directional signaling in PCP.

## RESULTS

### Pk and Sple do not bias microtubules in the D-wing

**Fig. 1.**
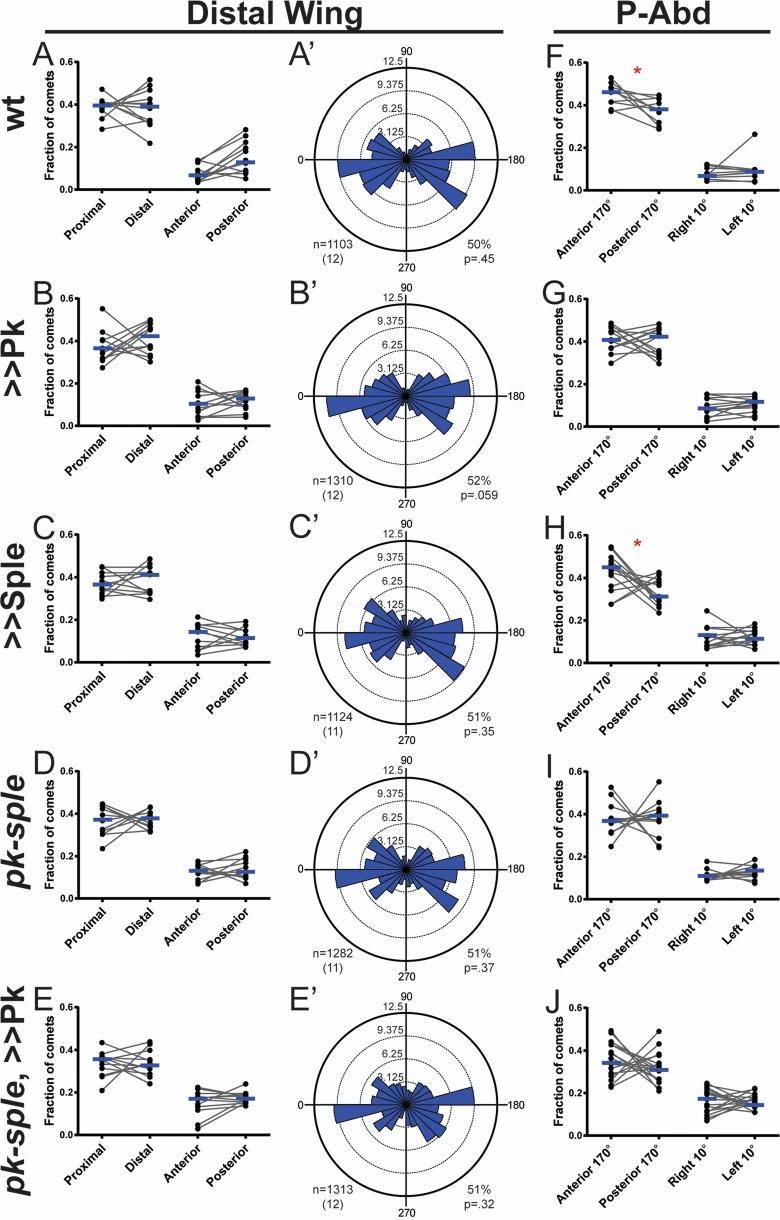
**Microtubule polarity in the D-wing and P-abd.** (A-E′) The fraction (A-E) or percentage (A′-E′) of Eb1::GFP comets observed moving in a given direction in wild-type (A, *P*=0.8984; A′); Pk-overexpressing (B, *P*=0.3013; B′); Sple-overexpressing (C, *P*=0.7578; C′); *pk-sple* mutant (D, *P*=0.7646; D′); and *pk-sple* mutant, Pk-overexpressing (E, *P*=0.8057; E′) D-wings. Genotypes for A,A′-E,E′ are numbers 1-5, respectively (see Materials and Methods). (F-J) The proportion (fraction) of Eb1::GFP comets observed moving in a given direction in wild-type (F, *P*=0.0273, 1808 comets from *n*=10 flies); Pk-overexpressing (G, *P*=0.7354; 1636 comets from *n*=13 flies); Sple-overexpressing (H, *P*=0.0398; 2131 comets from *n*=13 flies); *pk-sple* mutant (I, *P*=0.9097; 1839 comets from *n*=12 flies); and *pk-sple* mutant, Pk overexpressing (J, *P*=0.4233; 2184 comets from *n*=18 flies) P-abds. Genotypes for F-J are numbers 6-10, respectively. For A-E,F-J: grey lines link values from the same fly; blue bars mark the median; *P*-values are from a Wilcoxon matched-pairs signed rank test; *P<0.05. For A′-E′: *n* is the total number of comets with the number of flies is in parentheses; percentage is the proportion of comets moving towards the distal quadrant versus the proximal quadrant of angles; *P*-values are from a one-tailed binomial test. >> denotes overexpression.

We and others have previously observed that controlling the expression of Pk or Sple isoforms determines the direction of hair growth in almost the entire wing (Ayukawa et al., 2014; Gubb et al., 1999; Lin and Gubb, 2009; Olofsson et al., 2014; Strutt et al., 2013); however, we observed the microtubule polarity bias thought to underlie this directional control only in the P-wing (Harumoto et al., 2010; Olofsson et al., 2014). Microtubules in the D-wing, though in a largely parallel orientation, showed no polarity bias in either direction (Harumoto et al., 2010). We therefore asked if altering Pk or Sple isoform expression could introduce a plus-end bias in D-wing microtubules. Using Eb1::GFP to track the growing plus-ends of microtubules, we confirmed prior observations (Harumoto et al., 2010) that in the D-wing, microtubules grow largely along the proximodistal axis, yet their plus-ends are not biased in either the proximal or distal direction (Fig. 1A,A′; Fig. S1C,E,E′). As Pk is the dominant isoform in wing (Gubb et al., 1999; Lin and Gubb, 2009; Olofsson et al., 2014), we thought that perhaps a distal bias might be detectable upon overexpression of Pk. However, when we did this, microtubule polarity was unaffected (Fig. 1B,B′). We then overexpressed Sple and found that, while this reverses the direction of hair growth (Olofsson et al., 2014), microtubule polarity was unchanged (Fig. 1C,C′). Similarly, microtubule polarity was unaffected in D-wings mutant for both the Pk and Sple isoforms (*pk-sple* mutants, Fig. 1D,D′) as well as in *pk-sple* mutant wings in which Pk was overexpressed (Fig. 1E,E′). Thus, while Pk and Sple control the direction of hair growth in the entire wing, microtubule polarity in the D-wing is unaffected.

### Pk and Sple do not bias microtubules in the P-abd

In the dorsal P-abd, Pk and Sple control the direction of hair growth as they do in the wing and A-abd. Pk is dominant in this tissue, and overexpression of Sple reverses the direction of hair growth (Lawrence et al., 2004; Olofsson et al., 2014). Additionally, Ds in this tissue is high anteriorly and low posteriorly, and hairs grow from the posterior sides of cells such that, like in the Pk-dominant wing, hairs grow towards the low end of the Ds gradient (Casal et al., 2002). We therefore expected that if microtubule plus-ends were biased in this tissue that, like in the P-wing, they would be biased towards the low-end of the Ds gradient with more microtubule plus-ends growing towards the posterior sides of cells. If, conversely, microtubules behave as they do in the D-wing, they might display no bias. We found, however, that unlike either region of the wing, microtubule plus-ends were moderately biased, but towards the anterior sides of cells, opposite to where Fz and Dsh accumulate (Fig. 1F; Fig. S1F,F′). We then asked if overexpressing Sple, which reverses hair direction, would reverse the polarity bias of these microtubules and found that it had no effect on microtubule polarity (Fig. 1H). Furthermore, in Pk overexpressing; *pk-sple* mutant; and *pk-sple* mutant Pk overexpressing P-abds microtubule polarity was not consistently biased in either the proximal or the distal direction (Fig. 1G,I,J). Therefore, despite the robust effect in the P-abd of Pk and Sple on hair direction, they have at most a small effect upon microtubule polarity, and this effect is not consistent with the direction of hair growth. We therefore propose that, in the P-abd, Pk and Sple control the direction of hair growth through a microtubule-polarity independent mechanism.

### Dsh vesicle movement is biased distally but numbers of vesicles are very low

**Fig. 2.**
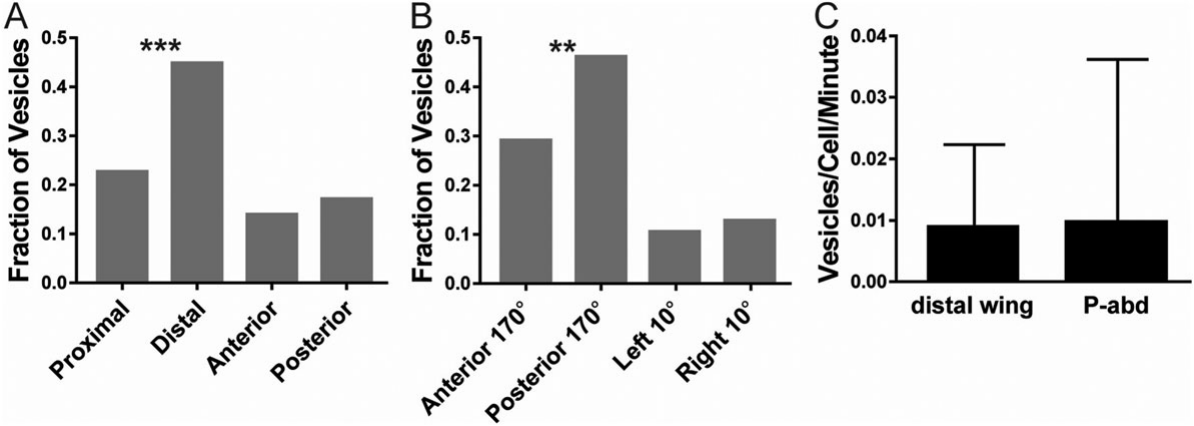
**Dsh vesicles in the D-wing and P-abd.** (A,B) Proportion (fraction) of Dsh::GFP vesicles observed moving in a given direction in D-wing (A, 126 total vesicles from 32 flies; *P*=0.00080; *n*=86 proximal and distal vesicles) and P-abd (B, 129 total vesicles from 56 flies; *P*=0.0098; *n*=98 proximal and distal vesicles). *P*-values from a one-tailed binomial test. (C) Average number of vesicles per cell per minute in each fly in the D-wing and P-abd. Error bars are s.d. **P<0.01; ***P<0.001. This figure uses genotype 14.

It is proposed that the microtubule-polarity bias in the P-wing and A-abd is important for PCP because the bias in microtubule-polarity introduces a bias in the direction of Fz and Dsh vesicle trafficking (Matis et al., 2014; Olofsson et al., 2014; Shimada et al., 2006). We therefore looked at the movement of Dsh vesicles in the D-wing and P-abd to see if, despite the absence of the predicted distal/posterior bias in microtubule polarity, vesicles move towards the distal/posterior sides of cells where they asymmetrically localize. While in both these tissues we observed that statistically significantly more Dsh::GFP vesicles moved towards the distal (D-wing) or posterior (P-abd) sides of cells (Fig. 2A,B), we observed ∼9-fold fewer vesicles in the D-wing and P-abd as compared to the P-wing [∼0.9 vesicles/cell/min (Olofsson et al., 2014) in P-wing compared to ∼0.01 in D-wing and P-abd] (Fig. 2C). In addition, vesicles that moved all the way from one side of a cell to the other were a very small fraction of all observed vesicles. Finally, when Sple was overexpressed in these tissues, virtually no vesicles at all were seen moving in cells. We conclude that while we cannot completely rule out a role for vesicle trafficking in providing a directional input in the D-wing and P-abd, it seems unlikely to be the dominant mechanism.

### *ft* mutant phenotypes in the abdomen

**Fig. 3.**
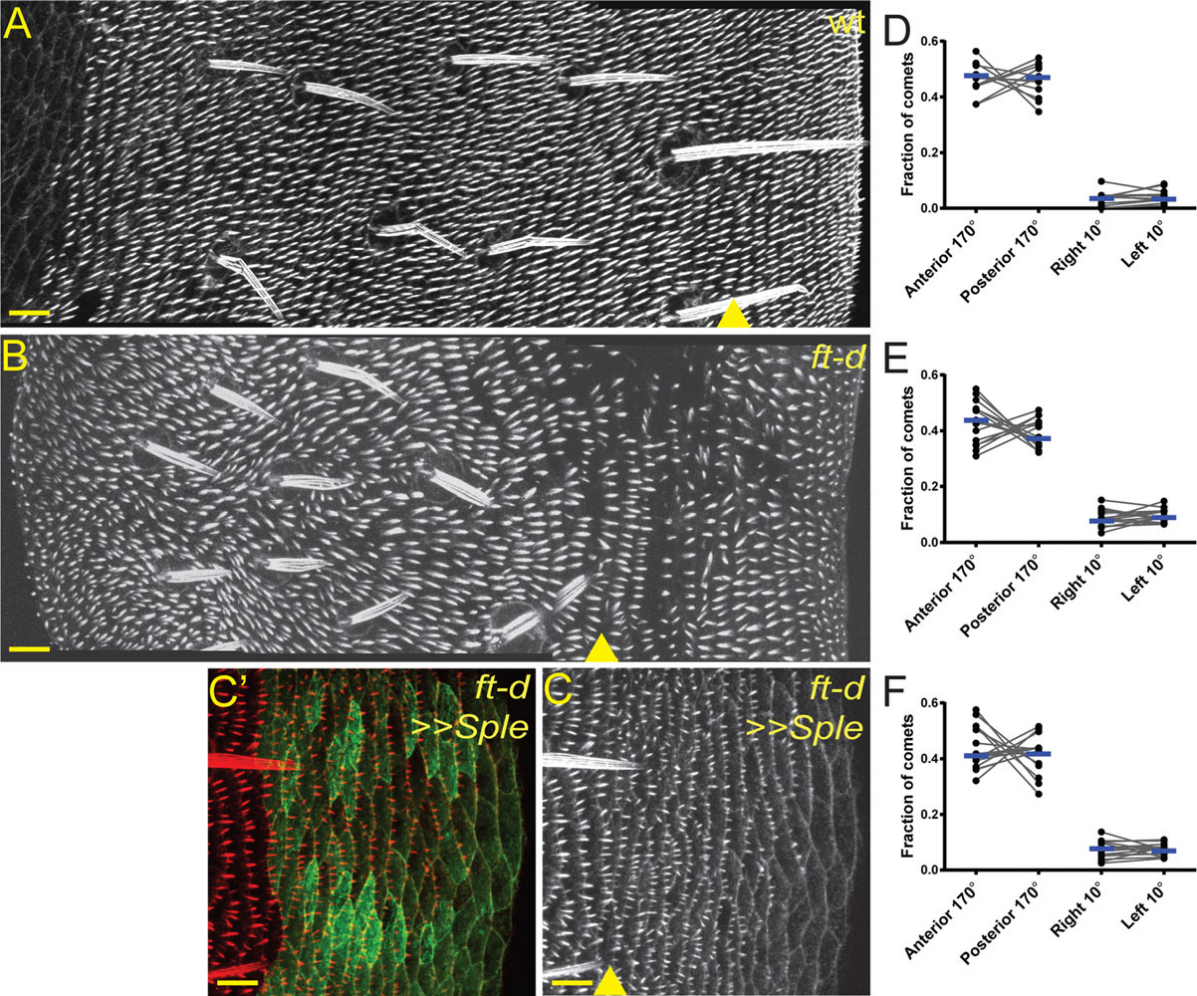
**Direction of hair growth and microtubule polarity in *ft, d* mutant abdomens.** (A-C′) Abdominal segments from wild-type (A, genotype 17), *ft-d* null (B, genotype 18), and *ft-d* null, overexpressing Sple (C,C′, genotype 12) flies stained with phalloidin to visualize actin-rich prehairs. Yellow triangle indicates approximate location of anterior-posterior compartment boundary. HhGal4-driven UAS-Eb1::GFP expression marks the posterior compartment in C′. Scale bar=10 µm. >> denotes overexpression. (D-F) The proportion (fraction) of Eb1::GFP comets observed moving in a given direction in *ft, d* mutant A-abds (D, *P*=0.9697; 3471 comets from *n*=12 flies; genotype 13); *ft, d* mutant P-abds (E, *P*=0.3591; 2091 comets from *n*=15 flies; genotype 11); and *ft, d* mutant, Sple overexpressing P-abds (F, *P*=0.8160; 2077 comets from *n*=15 flies; genotype 12). Grey lines link values from the same fly; blue bars mark the median; *P*-values are from a Wilcoxon matched-pairs signed rank test.

If tissue polarity is not dependent upon a microtubule-polarity bias or vesicle trafficking in these tissues, we then wondered what role, if any, Ft-Ds-Fj might play in determining polarity. Assaying PCP phenotypes in *ft* mutants is complicated by Ft's role in Hippo signaling, causing imaginal discs to overgrow and become tumorous when *ft* is mutated. The overgrowth phenotype is blocked by suppressing Hippo pathway activation by mutating *dachs* (*d*) in addition to *ft*. The direction of hair growth in *d* mutant wings is mostly normal, and Sple overexpression still reverses the direction of hair growth in this mutant background though the reversal of wing margin bristles is incomplete (Fig. S2). We have previously assayed microtubule polarity in the P-wing of *ft, d* double mutant flies and found that the microtubules are no longer organized in a parallel network (Olofsson et al., 2014). This correlates with several reports showing that when *ft* is mutated, large swirls of hairs are seen in the P-wing (Brittle et al., 2010; Matakatsu and Blair, 2006; Matis et al., 2014) (see Discussion for an alternative hypothesis to explain this phenotype). We then examined the A-abd of *ft, d* mutant flies because in the A-abd – like the P-wing – the direction of hair growth correlates with the plus-end microtubule polarity bias established by Pk and Sple. We found that in *ft, d* mutant A-abds the direction of hair growth was moderately disrupted (Fig. 3A,B) (Mao et al., 2006). In addition, we found that the posterior microtubule plus-end bias (Olofsson et al., 2014) was lost in *ft, d* mutant A-abds (Fig. 3D). This data is consistent with the mechanistic model that was proposed for the P-wing. Notably, it also suggests that in the absence of a directional signal from Ft-Ds-Fj, an unknown signal is present to weakly promote a posterior polarity.

Interestingly, the direction of hair growth in the P-abd was largely reversed in *ft, d* mutant flies (Fig. 3A,B). The boundary of this reversal did not line up precisely with the anterior-posterior compartment boundary (one to three cells posterior to the last row of bristles, defined by the ciGal4 and HhGal4 drivers, for schematic see Fig. S1B). Rather, the first one to three rows of cells in the P-abd grew hairs that pointed posteriorly while the rest of the P-abd cells grew hairs that pointed anteriorly (Fig. 3B). Interestingly, overexpressing Sple in the *ft, d* null P-abd reverted the direction of hair growth back to the wild type, posterior direction (Fig. 3C,C′). And while in *ft, d* mutant P-abds, microtubule-polarity was mildly disrupted compared with wild type (compare Fig. 1F with Fig. 3E), this disruption was not affected by overexpressing Sple (Fig. 3F). Thus, in the absence of signaling from the Ft-Ds-Fj system, Pk and Sple still control the direction of hair growth without affecting microtubule polarity, perhaps in response to another signal that is normally overpowered by signals from Ft-Ds-Fj in wild-type tissue. Whether this additional signal is the same as that which provides information to cells in the D-wing remains unknown.

## DISCUSSION

In one model for PCP signaling, supported by evidence from the P-wing and A-abd, anchored, parallel, apical microtubules support the directional trafficking of core PCP proteins Fz and Dsh (Harumoto et al., 2010; Matis et al., 2014; Shimada et al., 2006). The polarity of these parallel microtubules is differentially biased by Pk and Sple, thereby biasing the directional movement of the Fz- and Dsh-containing vesicles that travel along them (Olofsson et al., 2014). The bias in vesicle trafficking produces a slight increase in Fz and Dsh concentration on one side of the cell, and this imbalance is then amplified by the core module. This model not only suggests a cell biological link between the Ft-Ds-Fj system and the core module, but also provides a way, via expression of Pk or Sple, that tissues can differentially interpret Ds and Fj gradients (summarized in Table 1). In Sple-dependent tissues, it has also been proposed that anchoring of Sple to Ds and/or D transduces directional information to the core PCP system (Ambegaonkar and Irvine, 2015; Ayukawa et al., 2014). Since Sple, but not Pk, binds Ds and D, this mechanism is only possible in Sple dependent tissues and could co-exist with the microtubule biasing mechanism. The data presented here demonstrate that while the microtubule polarization mechanism might transduce global directional signals in some Pk-dependent tissues, it is not the only such mechanism. In both the D-wing and the P-abd – both Pk-dependent tissues – Pk and Sple are able to determine the direction of tissue polarity, yet microtubule polarity in the two regions remains unaltered. Here we discuss mechanisms that could be working in each tissue.

**Table 1.**
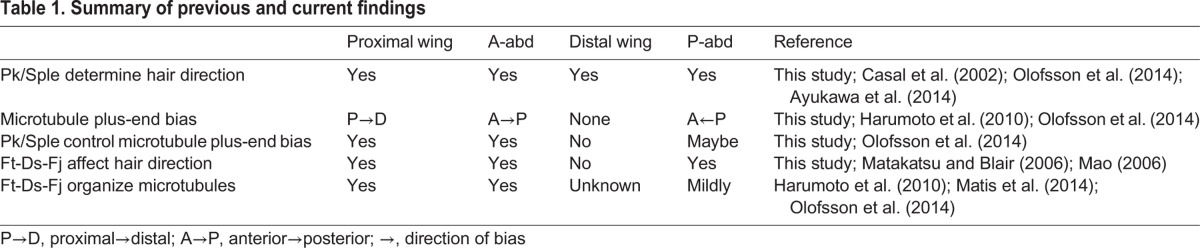
**Summary of previous and current findings**

Directional information in the wing appears to derive from at least two distinct signals (Sagner et al., 2012). In the P-wing, polarized apical microtubules direct distal trafficking of Fz and Dsh to orient core PCP polarization. When this signal is disrupted by mutation of *ft* or *ds*, the presence of an additional signal originating at the distal wing margin is revealed that maintains distal polarity in the D-wing (Brittle et al., 2010; Matakatsu and Blair, 2006; Matis et al., 2014). This distal signal has been proposed to result from the redundant action of Wnt4 and Wg expression at the margin (Wu et al., 2013). Because Pk and Sple determine the direction of hair growth in the D-wing despite their inability to alter microtubule polarity, Pk and Sple appear to interpret the margin signal by a microtubule-independent mechanism.

The combination of the proximal polarized-microtubule signal and a marginal Wnt4 and Wg signal could work in a partially overlapping fashion to provide directional information throughout the wing, a model that requires more rigorous testing by examining flies in which both the Ft-Ds-Fj and Wnt4/Wg systems are compromised. This is technically very difficult as the genes for all but Fj are located on the left arm of the second chromosome. Furthermore, mutating *ft* or *wg* causes confounding phenotypes that must be mitigated to facilitate examining polarity. Finally, a more definitive understanding of how Pk and Sple are altering tissue polarity in the D-wing will require a better understanding of the mechanism of any distal or margin derived directional signal.

In the dorsal P-abd, a Pk-dependent compartment, our data show that Pk and Sple must determine the direction of tissue polarity through a microtubule-polarity independent mechanism. Microtubule polarity is not as expected in the wildtype P-abd; additionally, reversal of the orientation of core module and hair polarity (Lawrence et al., 2004; Olofsson et al., 2014) is unaccompanied by reversal of microtubule polarity in P-abd (Fig. 1F-J). Our data also show that Pk and Sple determine direction downstream of multiple directional cues: the absence of Ft reveals a cryptic directional signal that directs an anterior polarity in most of the posterior compartment (Fig. 3B). Similar observations have been made in a *ds* hypomorph (Casal et al., 2002), and a similar cryptic signal has also been proposed in P-compartment of the ventral abdomen (Ambegaonkar and Irvine, 2015). We observe that the interpretation of this cryptic signal is altered by overexpressing Sple in the *ft, d* mutant background, reverting the polarity direction to the posterior (Fig. 3C,C′). The mechanisms through which Pk and Sple not only control microtubule polarity but also interpret signals from this unknown cryptic source are not clear.

As an alternative, microtubule-independent mechanism, Sple has been shown to bind to Ds and/or D, tethering it to one side of the cell, thereby introducing a bias to Sple localization that is expected to orient core PCP polarization in Sple-dependent tissues (Ambegaonkar and Irvine, 2015; Ayukawa et al., 2014). The contribution from this mechanism and from a mechanism based on microtubule polarity and vesicle trafficking need not be mutually exclusive. In Pk-dependent tissues, an analogous mechanism would predict that Pk is tethered to the opposite side of the cell, since the relationship between core PCP and Ds and Fj gradients is reversed. Whether analogous binding occurs between Pk and Ft is unknown. In contexts in which neither biased, microtubule-dependent trafficking nor Ft-Ds heterodimers are providing directional information (such as the *ft, d* mutant P-abd), tethering of Sple, Pk, or another core component to any other asymmetrically arrayed molecular cue could be a primary source of global directional information.

In the wing, it has been proposed that the parallel apical microtubules whose polarity is biased by Pk and Sple are organized by the Ft-Ds-Fj system because in *ft*, *ds* or *ft, d* double mutant cells the microtubule network is disorganized (Harumoto et al., 2010; Matis et al., 2014; Olofsson et al., 2014). This view has been challenged, in part due to the observed binding of Sple but not Pk to Ds and D, leading to the alternative suggestion that the core PCP system is directed by the Ft-Ds-Fj system by Sple tethering to Ds or D, but that core PCP is uncoupled from and not oriented by Ft-Ds-Fj in Pk-dependent tissues (Ambegaonkar and Irvine, 2015), an argument first proposed by others (Merkel et al., 2014). According to this model, *ft* and *ds* polarity phenotypes in Pk-dependent tissues are produced by interference from mislocalized Sple (Ambegaonkar and Irvine, 2015). However, several observations are difficult to reconcile with this model. Most persuasive is the observation that in the distal wing, where polarity is Pk-dependent, an artificially reversed gradient of Ds (induced by Dll-GAL4 driven expression of UAS-Ds) reverses polarity in the direction expected of a Pk-dependent response (Harumoto et al., 2010). This cannot be explained by Ds engaging and anchoring Sple, since that would promote hairs pointing distally. Another complication for the above model is the observation that Pk expression reverses polarity in the Sple-dependent A-abd (Olofsson et al., 2014). This implies that Pk is interpreting Ft-Ds-Fj signals in this tissue, or that there is another directional signal that can be interpreted by Pk. Based on these observations we therefore suggest that Ft-Ds-Fj influences Pk-dependent polarity without involving Sple coupling. A second argument is the observation that, in vertebrates, the Ft-Ds-Fj and core PCP systems both influence some of the same polarity phenotypes (Wallingford, 2012), yet there is no Sple ortholog in vertebrates to mediate interaction between these systems. We therefore think it is premature to dismiss the possibility that Ft-Ds-Fj provides input to Pk-dependent core polarization independent of Sple-mediated coupling.

Furthermore, our data argue that Sple can direct polarity by a mechanism independent of tethering by the Ft-Ds-Fj system. In the P-abd, we showed that *ft d* mutants display reversed polarity in a portion of the compartment, and this anterior polarity reverted to posterior polarity upon Sple overexpression. In this circumstance, D is absent and Ds is not expected to be polarized, implying that Sple is not acting by tethering to these components. Similarly, it was argued that in most of the wing disc, D directs Sple localization, whereas Ds is unable to do so because Ds expression is apparently too low in the distal wing (Ambegaonkar and Irvine, 2015) (see also Hogan et al., 2011; Ma et al., 2003; Matakatsu and Blair, 2004; Rogulja et al., 2008). Yet, we find that in a *d* mutant wing, Sple overexpression is still capable of reversing polarity (Fig. S2). Multiple groups have argued that Ds is involved in allowing Sple to control the direction of tissue polarity in the wing (Ayukawa et al., 2014; Hogan et al., 2011; Merkel et al., 2014), so perhaps sufficiently high levels of Sple allow interaction with the low levels of Ds. Alternatively, Sple may work through a different mechanism in this circumstance. We note that the direction of wing margin bristle growth in this *d* mutant case is altered but is not fully reversed. While we, and others, have observed that Pk and Sple isoform expression in the wildtype wing determines the direction of growth of both the hairs and the wing margin bristles (Ayukawa et al., 2014; Doyle et al., 2008; Gubb et al., 1999; Lin and Gubb, 2009; Olofsson et al., 2014), the mechanisms governing the direction of bristle growth are relatively unstudied as compared to those governing the direction of hair growth. It may be that differential contributions of D and Ds in hairs versus bristles determine the differential sensitivity to Sple overexpression.

While in the P-wing and A-abd, specifying the direction of polarity seems to depend upon control of microtubule polarity, we have shown that this is not true in all tissues. Pk and Sple control the direction of polarity in all observed tissues, yet cells of the P-abd depend on Ft but not a microtubule bias. In the D-wing, microtubule polarity is not affected by Pk or Sple expression, nor is a microtubule polarity bias observed, yet Pk and Sple control the direction of polarization, indicating a microtubule-independent mechanism. The D-wing and P-abd thus provide two additional signaling paradigms that can, going forward, be used for discovery of additional cell biological and signaling mechanisms important for PCP.

## MATERIALS AND METHODS

### Fly genotypes

Eb1::GFP comet assays in D-wing: (1) *ciGal4/UAS-Eb1::GFP*, (2) *UAS-pk/+; ciGal4/UAS-Eb1::GFP*, (3) *ciGal4/UAS-sple, UAS-Eb1::GFP*, (4) *pk-sple^13^/pk-sple^14^; ciGal4/UAS-Eb1::GFP*, (5) *pk-sple^14^, UAS-pk/pk-sple^13^; ciGal4/UAS-Eb1::GFP*.

In P-abd: (6) *HhGal4/UAS-Eb1::GFP*, (7) *UAS-pk/+; HhGal4/UAS-Eb1::GFP*, (8) *HhGal4/UAS-sple, UAS-Eb1::GFP*, (9) *pk-sple^13^/pk-sple^14^; HhGal4/UAS-Eb1::GFP*, (10) *pk-sple^14^, UAS-pk/pk-sple^13^; HhGal4/UAS-Eb1::GFP*, (11) *ft^8^, d^1^/ft^8^, d^GC13^; HhGal4/UAS-Eb1::GFP*, (12) *ft^8^, d^1^/ft^8^, d^GC13^; HhGal4/UAS-sple, UAS-Eb1::GFP*.

In A-abd: (13) *ft^8^, d^1^/ft^8^, d^GC13^; ciGal4/UAS-Eb1::GFP*.

Dsh::GFP vesicle tracking in D-wing: (14) *Dsh::GFP (II)*, (15) *D174Gal4; Dsh::GFP; UAS-sple/+*.

In P-abd: (14), (16) *Dsh::GFP; HhGal4/UAS-Sple*.

Phalloidin staining: (17) *OREGON-R*, (18) *ft^8^, d^1^/ft^8^, d^GC13^*, (12).

Adult wings: (19) *d^1^/d^GC13^; ciGal4/+*, (20) *d^1^/d^GC13^; ciGal4/UAS-sple*.

### Live imaging and analysis of Eb1::GFP and Dsh::GFP

Imaging and analysis of Eb1::GFP comets and Dsh::GFP vesicles was performed as per Olofsson et al. (2014). Briefly, movies were taken from 24 h after pupal formation (APF) (wing) or 42 h APF (abdomen) pupae using a Leica SP5 confocal microscope and angles of puncta trajectories were tracked using Fiji (Schindelin et al., 2012). For comet analysis, color-coded hyperstacks were used (Fig. S1D-F′). Graphs were generated using GraphPad Prism version 6.05 for Windows and Oriana4 (Kovach Computing Services). *P*-values were calculated with a one-tailed binomial test (*n*=number of comets/vesicles in all flies). *P*-values for comets were also calculated using the Wilcoxon matched-pairs signed rank test (*n*=number of flies) to analyze the consistency of a plus-end polarity bias from fly to fly. For region of wing where imaging was done, see Fig. S1C.

### Phalloidin staining

Pupae were aged to 45-47 h APF. Pupae were dissected, fixed and mounted as in (Wang and Yoder, 2011). Fixation was in 4% paraformaldehyde with 0.1% Triton X-100. Tissue was washed in PBS before staining with Alexa Fluor 488- or 594-conjugated phalloidin (Molecular Probes) and mounting in Vectashield mounting medium for fluorescence (H-1000, Vector Labs). *Z*-stacks were captured using a Leica SP5 confocal microscope. Images were processed in Fiji including image stitching using the MosaicJ plugin (Thévenaz and Unser, 2007).
